# Persuasive Capacity, Not Persuasive Power: A Commentary on Ari Deller’s “The Epistemic Costs of Super-Persuasive AI”

**DOI:** 10.1007/s13347-026-01156-8

**Published:** 2026-08-05

**Authors:** Robin McKenna

**Affiliations:** University of Liverpool, Liverpool, United Kingdom

**Keywords:** Persuasion, AI, AI persuasion, Epistemology

## Abstract

In a recent article in this journal, Ari Deller has argued that there are some severe epistemic costs to super-persuasive AI. In this commentary I explain why, while I agree with Deller about the epistemic costs of super-persuasive AI, I disagree with him about the likely source of the problem. Where Deller focuses on the super-persuasive power of future AI technologies, I focus on the enormous increase in persuasive capacity that is provided by a technology that can produce very large amounts of persuasive content at minimal cost.

In “The Epistemic Costs of Super-Persuasive AI” Ari Deller argues for three claims:

1. While large language models (LLMs) will increasingly be optimised for persuasion, to the point where their persuasive capabilities far exceed our own, it is less likely that they will be optimised for truth. This is not primarily because of malign intentions on the part of AI companies; rather, while we have the capacity to optimise for persuasiveness (we can tell when we are persuaded) we often lack the capacity to evaluate for truth because we will increasingly not know enough or have the cognitive abilities required to make the necessary evaluations (Deller 2026, 2–7).
2. Because LLMs will be optimised for persuasion rather than truth, they will get increasingly good at producing persuasive arguments for any conclusion whatsoever, including infelicitous ones, where an argument is infelicitous if its conclusion is false, or it doesn’t provide good reasons for believing the conclusion. The result is that an argument’s being persuasive ceases to be a good indication that it is a good (felicitous) argument (Deller 2026, 7–12).
3. This calls for regulatory curbs on the production and deployment of persuasive AI, together with technical work on “truth-aligned” systems (Deller 2026, 19–20).

I am sympathetic to (2), in particular Deller’s claim that beliefs formed on the basis of LLM-generated arguments are subject to a defeater, and so they cannot amount to knowledge even when true (Deller 2026, 8–12). Because—Deller assumes—we will increasingly form beliefs on the basis of LLM-generated arguments, this threatens a very wide range of beliefs. But I think a better case can be made for this conclusion if we replace (1) with a different claim: what matters is not that LLMs may soon have substantially greater per-unit persuasive power than humans, but that they can generate persuasive arguments on all sides of any question at negligible marginal cost, and so in numbers that our argumentative practices cannot hope to absorb (McKenna, 2026).

## An Alternative Argument for (2)

Deller does *not* argue that, because an LLM-produced argument can be persuasive yet infelicitous, we have a standing undercutting defeater for any beliefs produced via LLM-generated arguments. This would be a variant on the standard Cartesian sceptical argument, which appeals to the possibility of error to motivate a sceptical conclusion (Deller, 2026, p. 11). He argues that, because LLMs are optimised for persuasion rather than truth, they will produce a large number of persuasive yet infelicitous arguments—arguments that succeed in getting people to form new beliefs even though their conclusions are false or they do not offer good reasons for their conclusions. The result is that a large proportion of the “stock” of persuasive arguments that we use to form our beliefs will be infelicitous (Deller, 2026, p. 10). It is this that provides the undercutting defeater: the problem is not that we might, for all we know, be persuaded by infelicitous arguments but that the stock of persuasive arguments is contaminated in such a way that persuasiveness no longer indicates felicity. -

We need to distinguish between two ways in which the stock of persuasive arguments could become contaminated. One way—what Deller has in mind—is that LLMs get increasingly good at producing persuasive arguments for any conclusion whatsoever, including infelicitous ones—they become “super-persuasive”. The problem is the result of large increases in the per-unit persuasive *power* of LLM-generated arguments; they are far more persuasive than arguments produced by humans. Another way—which is explored in McKenna (2026)—is that, while there may be some increase in the per-unit persuasive power of LLM-generated arguments, this will be swamped by enormous increases in the *quantity* of LLM-generated arguments that are available for general consumption. The problem is not per-unit persuasive power but persuasive *capacity.*

Let me develop this alternative in more detail. Imagine that LLMs are, in the near future, widely used to produce arguments across the contested domains that Deller focuses on—politics, ethics, and so on. Two things seem plausible in this scenario. First, these technologies will be deployed by actors with a variety of commitments on the issues in question. Second, because the “cost” of producing arguments using LLMs is extremely low, at least in comparison to the cost of commissioning a human to produce them, we will end up with an extremely large number of arguments—far more than we would have without LLMs. Consequently, we will end up with a huge number of arguments for mutually incompatible conclusions. Because the conclusions are mutually incompatible, many of these arguments will not be felicitous.

For this to happen, LLM-produced arguments would need to be persuasive. But they would simply need to be at least as persuasive as high-quality human-produced arguments. A large increase in quantity is enough because, if there are lots of arguments to consider, it will simply be impossible for anyone to subject more than a few of them to proper scrutiny. Without getting into the tricky issue of how to count or individuate arguments, it seems safe to assume that LLMs will be able to produce far more arguments, on all sides of an issue, than any human could ever properly scrutinise.

To put it somewhat metaphorically, the “space” or “marketplace” of arguments will increasingly be dominated by LLMs, crowding out humans due to economies of scale. Just as a company can dominate a marketplace not by producing a superior product but by producing more of a standard product than their competitors at a lower price, LLMs can dominate the “marketplace of arguments” without the capacity to produce super-persuasive arguments.

## Quantity Over Quality

Why should we prefer my argument to Deller’s? Let me give four reasons.

The first is that Deller’s argument is a hostage to fortune; it relies on the future development of LLMs—they will, in the relatively near future, be super-persuasive. My argument is not a hostage to fortune. Indeed, you can run my argument based on what we know about the persuasive capabilities of the current generation of LLMs. As Deller notes in various places, the current generation is already at least as persuasive as humans (see, for example, Bai et al., 2023).

The second reason is related to one that Deller discusses. Deller’s argument relies on scaling laws—AI development follows a relatively predictable path, with regular improvements due to increased compute and improved efficiency. Let’s assume, at least for the sake of argument, that this is true of a wide range of cognitive tasks (Deller, 2026, 3–4). But persuasion is not a purely cognitive task; it depends on uptake from the intended audience—the people you are trying to persuade. While Deller acknowledges there may be limits on how persuadable humans are due to an evolved capacity for epistemic vigilance (Sperber et al., 2010), he doesn’t take this possibility to be a problem for his argument (Deller, 2026, 14–15). His basic response is this: either epistemic vigilance is consistent with being persuaded by LLM-produced arguments, in which case the original argument goes through, or it takes an extreme form which leads to the rejection of felicitous persuasive arguments as well as infelicitous ones, in which case we get a sceptical result via other means.

Another possibility is worth considering: while LLM-produced arguments can bypass our mechanisms for epistemic vigilance, this will often not be a function of their persuasive power but of the sheer amount of arguments that can be produced. That is, LLMs have the potential to *overwhelm* these mechanisms, and so to achieve large persuasive effects not via an increase in per-unit persuasive power but via an increase in quantity. As with my alternative to Deller’s original argument, this has the advantage of avoiding speculation about the future persuasive power of LLMs. It has the additional advantage of not requiring any revisions to our basic understanding of epistemic vigilance. Even evolutionarily adaptive mechanisms can be overwhelmed by a quantity of inputs they did not evolve to cope with.

The third reason is based on an analogy Deller draws in support of his own argument. He compares our situation with respect to LLM-produced arguments with thermal testing to distinguish diamonds from fakes. Thermal testing worked as a means of identifying genuine diamonds until moissanite began to be mass-produced in the 1990s. The problem though is not just that moissanite passes these tests. The problem is that there was a lot of it around, so existing tests were no longer a reliable way to distinguish diamonds from fakes. Deller thinks something similar will soon apply to arguments: whatever tests we have been using to distinguish felicitous from infelicitous persuasive arguments, they will no longer work in an age of super-persuasive AI.

But the problem is not just that moissanite passes these tests but that it is mass-produced. What this shows is that, when you have a process for distinguishing between the genuine article (a diamond, a felicitous argument) and a fake (moissanite, a persuasive but infelicitous argument), problems result as soon as you have a large number of fakes that can pass the test. It does not matter *how well* they can pass the test. Deller himself seems to recognise this. He writes: “What did undermine the usefulness of thermal testing was the saturation of the market with moissanite, because such saturation undercut the principle that high thermal conductivity is indicative of a diamond’s authenticity” (Deller, 2026, p. 11). But he does not draw the obvious conclusion, which is that saturation is itself enough to generate the defeater, provided the fakes pass the test. The upshot is that, at least so far as Deller’s argument is concerned, it does not matter *how* persuasive LLM-generated arguments are, so long as they can pass some threshold for persuadability, and there are a lot of them around.

Finally, it is interesting to consider whether Deller’s argument overgeneralises in the following way. Intellectuals are good at producing arguments that are persuasive, at least to some audiences, but infelicitous. This claim should not be controversial: it follows from the simple fact that intellectuals argue for mutually incompatible conclusions, so it must be that a fairly large proportion of their arguments are infelicitous in Deller’s sense. Can we then conclude that intellectual-generated arguments are subject to an undercutting defeater and cannot yield knowledge?

I assume Deller does not want to accept this conclusion. But—at least as far as I can see—his best way of resisting it is to appeal to the comparative *scarcity* of intellectual-generated argumentation. Persuasive arguments are *difficult for us to produce* (Deller, 2026, p. 10). Human argument-production is slow, effortful and rate-limited. What keeps the marketplace of intellectual-generated arguments relatively healthy then is a production constraint. But, if this is right, then the disanalogy between intellectual-generated arguments and LLM-generated arguments is quantitative rather than qualitative, as I have been suggesting.

## Why Does it Matter?

When it comes to the practical upshots, does it matter whether we adopt Deller’s argument or my alternative? Good solutions to a problem are often still good solutions even if they are based on a misdiagnosis. I am though a little concerned that Deller’s remedies are quite small-scale, given the size of the problem he describes. He proposes a combination of regulations and attempts to fix the problem that LLMs optimise for persuasiveness, not truth. But if we view the problem as I have suggested—as one of over-production, and the saturation of the marketplace—it is hard to see how one could tackle the problem by placing regulations on the product or on deployment of the technology. The problem is one of market domination, and we should be suspicious of the idea that you can solve the problem of market domination via regulatory mechanisms.

## Data Availability

Not applicable.
